# In this month’s Bulletin

**DOI:** 10.2471/BLT.22.000922

**Published:** 2022-09-01

In the editorial section, Yot Teerawattananon et al. (526) point out that future procurement of vaccines for COVID-19 needs to account more accurately for the doses that can be delivered. Shaoshan Liu et al. (527) explore the potential of autonomous mobile clinics for provision of services in rural and remote areas.

Vijay Shankar Balakrishnan (530–531) reports on investigations into a current outbreak of acute hepatitis in children and how this process may prompt research on other causes. Balakrishnan interviews Yōhei Sasakawa (532–533) about his work in global health philanthropy with a focus on leprosy.

## Kenya 

### Is it possible for people to comply with control measures?

Heather R Chamberlain et al. (562–569) propose a method to estimate the feasibility of physical distancing.

## Mozambique

### How safe is the drinking water?

Hirotsugu Aiga et al. (534–543) examine microbial contaminants of water from improved sources.

## Nepal, South Africa

### The effects of examining distress

Raquel Burgess et al. (578–580) draw attention to the need to support research staff.

## Global

### Misleading, incorrect and voluminous 

Israel Júnior Borges do Nascimento et al. (544–561) review evidence of an infodemic.

### Taxes on alcohol, tobacco and sugar-sweetened beverages

Erika Siu and Anne Marie Thow (570–577) show examples of improved earmarking of taxes for health.

